# Exploring Effects of Protease Choice and Protease Combinations in Enzymatic Protein Hydrolysis of Poultry By-Products

**DOI:** 10.3390/molecules26175280

**Published:** 2021-08-31

**Authors:** Diana Lindberg, Kenneth Aase Kristoffersen, Sileshi Gizachew Wubshet, Linn Maria Gundersen Hunnes, Marte Dalsnes, Katinka Riiser Dankel, Vibeke Høst, Nils Kristian Afseth

**Affiliations:** Nofima AS, Osloveien 1, 1433 Ås, Norway; Diana.Lindberg@Nofima.no (D.L.); Kenneth.Kristoffersen@Nofima.no (K.A.K.); Sileshi.Wubshet@Nofima.no (S.G.W.); linnmaria.g.hunnes@gmail.com (L.M.G.H.); m.dalsnes@gmail.com (M.D.); Katinka.Dankel@Nofima.no (K.R.D.); Vibeke.Host@Nofima.no (V.H.)

**Keywords:** enzymatic proteins hydrolysis, stem Bromelain, Endocut-02, SDS-PAGE, SEC, poultry by-products, protease combinations, mechanically deboned chicken residue

## Abstract

A study of the effects of single and combined protease hydrolysis on myofibrillar versus collagenous proteins of poultry by-products has been conducted. The aim was to contribute with knowledge for increased value creation of all constituents of these complex by-products. A rational approach was implemented for selecting proteases exhibiting the most different activity towards the major protein-rich constituents of mechanically deboned chicken residue (MDCR). An initial activity screening of 18 proteases on chicken meat, turkey tendons and MDCR was conducted. Based on weight yield, size exclusion chromatography (SEC) and SDS-PAGE, stem Bromelain and Endocut-02 were selected. Studies on hydrolysis of four different poultry by-products at 40 °C, evaluated by protein yield, SEC, and SDS-PAGE, indicate that the proteases’ selectivity difference can be utilized in tailor-making hydrolysates, enriched in either meat- and collagen-derived peptides or gelatin. Three modes of stem Bromelain and Endocut-02 combinations during hydrolysis of MDCR were performed and compared with single protease hydrolysis. All modes of the protease combinations resulted in a similar approximately 15% increase in product yield, with products exhibiting similar SEC and SDS-PAGE profiles. This shows that irrespective of the modes of combination, the use of more than one enzyme in hydrolysis of collagen-rich material can provide means to increase the total protein yield and ultimately contribute to increased value creation of poultry by-products.

## 1. Introduction

The value creation of side streams and by-products from food processing sectors, collectively called by-products, is one of the global efforts to sustainably utilize marine and agricultural resources. These by-products contain proteins with potentially bioactive properties, eligible for recycling and upgrading for higher-value products, e.g., for human, pet food and feed purposes. If aiming at the feed, pet food, and human markets, strict regulations exist, governed by for example European Union (EU) regulations. Furthermore, if following existing food regulations and provided the materials are not included in the animal by-product categories, there are many industrial by-products that can be used for human consumption [1]. However, despite the successful developments of a few high-value products, especially animal-based by-products remain underutilized or utilized to applications with low value [1,2]. 

In recent years, enzymatic protein hydrolysis (EPH) has gained significant attention as a sustainable and versatile processing technology to extract and valorize proteins from animal and marine industrial by-products [1]. During the EPH process, proteases are added to the substrate, e.g., by-products, to facilitate both solubilization of proteins and their further breakdown into smaller peptides and free amino acids. After protease inactivation at near-boiling temperature, the EPH process often includes a downstream recovery of three crude fractions: solubilized peptides, lipids, and a collagen- and mineral-rich low-value sediment. The amount of proteins liberated from raw materials during EPH are fundamentally dependent on the composition and pre-treatment of the raw material, choice of protease and processing conditions [3,4,5].

One industrial by-product with high potential for valorization for human consumption is the remaining carcass after mechanical deboning of poultry, containing both meat with high nutritional value as well as high amounts of connective tissues and bones [4,6]. Collagen peptides have recently attracted much attention due to properties such as neutral odor, emulsification and stabilization, low allergenicity as well as antioxidant and antimicrobial activity [7]. However, while the myofibrillar proteins in meat are easily solubilized during EPH, collagenous proteins in cartilage or bone structures are harder to solubilize, mainly due to the more inaccessible collagen structure [6,7,8]. This is partly caused by the lack of sites within the repetitive sequence of amino acids, involving specific amino acids, that correspond to the selectivity of commercial proteases. The peptide chains also form a closely packed triple-helical structure, which in turn forms highly stable collagen fibers forming a tight network in tendons and bones, further reducing the possibility for proteases to bind to and hydrolyze single peptide bonds [9]. Hence, to be able to maximize the overall yield and tailor-make protein products of specific properties, it is important to develop strategies on how to solubilize not only the meat-derived proteins but also collagen-derived proteins from poultry carcasses. 

Previous studies have investigated protein extraction from mechanically deboned poultry by-products using commercial proteases. These studies are often focusing on achieving the best possible overall protein yield or achieving specific product properties, e.g., the highest possible bioactivity or the most neutral sensory attributes. This is usually done by selecting one or a limited number of proteases to reach the goal of the study [10,11,12]. A rationale for selecting these proteases is seldom provided, although information about protease substrate selectivity and activity arguably would enhance the chance of a successful outcome. One exception to this was a study by Nikolaev et al., where a rational approach was used aiming at producing a high yield poultry-based product containing peptides with low allergenicity and bitterness [13]. To be able to find the most optimal proteases able to reduce the allergenicity of major allergenic proteins, the specificity of available commercial proteases was in-silico analyzed and compared to preferred hydrolytic sites in the main antigens of the allergenic proteins in broiler necks. Based on this, four commercial proteases were selected for further lab-based optimization studies.

A feasible route to produce protein hydrolysates with tailor-made properties from poultry by-products is by using raw materials with higher amounts of one or the other of the components (e.g., after sorting or pretreatment). Aiming at developing a product consisting of mainly collagen peptides with anti-inflammatory activity from spent hens, Offengenden et al. employed a stepwise processing regime [14]. To reduce the amount of meat, it was manually removed from spent hens. The rest was homogenized with water and sieved, and subsequently, the retentate was treated with an acid solution to remove soluble proteins and fat. In the second step, EPH was performed on the collagenous acid-treated retentate with either one or two proteases in a consecutive manner. In other studies, acid or alkali treatment has been used for the removal of meat proteins from complex collagen-rich by-products. However, acid or alkali treatment is known to lower the nutritional value of proteins [15]. Consequently, the use of such approaches for removing meat from collagen material can be considered non-optimal for value-creation from both a sustainability and a circular economy perspective and one should rather strive for maximum value creation of all inherent components in raw materials.

The aim of the current study was to evaluate how product properties of poultry by-product hydrolysates were affected: (1) by choice of protease; and (2) by combining two proteases showing high activity towards either collagenous or myofibrillar proteins, respectively. A rational approach for finding the proteases that could maximize the yield of both proteins was conducted. The selectivity and effects on product properties were evaluated using a total of 18 proteases in EPH of five industrially relevant sources of poultry by-products (i.e., turkey tendons (TT) or turkey collagen tissue (TCT), minced chicken leg bones (CB), minced chicken meat (CM) and mechanically deboned chicken residues (MDCR)). Proteases were compared based on weight yield and product characteristics. A deeper study on the products resulting from two selected proteases was conducted based on laboratory-scale EPH on the same raw materials. Lastly, the products resulting from EPH combining the two proteases were evaluated based on hydrolysis yield and product characteristics. Furthermore, to investigate the feasibility of reducing the environmental load, a hydrolysis temperature close to the melting point of collagens of 40 °C was chosen. 

## 2. Results and Discussion

### 2.1. Protease Activity Screening

#### 2.1.1. Activity Determination with Azo-Casein Assay

It is well known that commercial protease mixtures exhibit different activities on different raw materials [3]. Therefore, to be able to normalize the amount of protease used in later steps involving investigations of substrate preference in the hydrolysis of different poultry raw materials, 18 proteases were subjected to a protease assay based on degradation of the synthetic substrate azo-casein (pH 7.0, 40 °C, 10 min). Casein has been successfully used to normalize protease activities in a study on salmon by-products [3]. Further, use of a neutral pH allows for an easy implementation of the methodology for by-product EPH processing plants working at these conditions. Curve fits based on absorbance after proteolysis spanning at least three different protease concentrations on one substrate concentration was obtained. The resulting straight-line curve fits (i.e., y = kx + m) were used to find the dilution of each protease at y = 1 (Table S1), which was set to the normalized activity. 

The investigated proteases exhibited measurable activities at these conditions, conforming to the generality of the assay. The lowest activities towards azo-casein, at current hydrolysis conditions, were seen using commercial proteases Maxipro NPU and Neutrase, and the highest using the commercial proteases stem Bromelain BR 1200 (Bromelain), Endocut-02, and Tail-10. To be able to reach the same azo-casein proteolysis rate, a 29-fold higher protease amount was required for the least active protease (Maxipro NPU), as compared to the most active one (Endocut-02). Two proteases were excluded in the subsequent poultry raw material screening due to their low activity with azo-casein, i.e., Maxipro NPU and Neutrase. For the other 16 proteases, the normalized protease activity, involving optimized amounts of each protease, was used in the subsequent substrate screening 

#### 2.1.2. Screening of 16 Proteases on Different Poultry Raw Materials

A small-scale screening of activity of the resulting 16 proteases on four different substrates were performed: hand-scraped turkey tendons, i.e., TT, as well as CB, CM and MDCR. The hydrolysis reaction was run for one and three hours using the same hydrolysis conditions as the initial protease screening with azo-casein. The weight yield-based hydrolysis efficiency was evaluated based on establishing the remaining amount of dried non-digested raw material after hydrolysis (Figure S1). For most of the proteases and substrates, the weight yield of samples after a total hydrolysis time of 3 h was higher than after 1 h. However, results indicate that 3 h of hydrolysis time yield yields little extra in terms of further hydrolysis of the raw materials. The increase in yield over time in protease hydrolysis normally levels off at a given point due to, for example, protease autolysis or heat-induced denaturation, substrate or product inhibition, or a decreased access to favorable substrate binding sites [16]. It is interesting to note, exemplified by e.g., Corolase 7090 and Protamex, that proteases rendering the high weight yield on collagen-rich materials TT and CB not necessarily resulted in the highest yield from the mixed collagen-rich MDCR material.

Large activity variations were observed between proteases on the four different substrates, as presented in Table 1, summarizing the weight yields after 1 h hydrolysis, as well as the TT/CM ratio for the 1 h EPH reactions.

As evident by ratio values close to 100%, proteases such as FoodPro PNL and Endocut-01 were as capable of digesting the CM material equally well as the collagenous TT material using the current hydrolysis conditions. Other proteases showed a significantly higher ability to digest one over the other material. The TT/CM-ratio was used as an initial guide for protease choice. However, to be able to select the two most complementary proteases out of the proteases exhibiting the highest and lowest TT/CM-ratios, further investigations into product characteristics were performed. A study of the peptide size distributions in the CM, CB, and MDCR samples hydrolyzed for 1 h was performed using SEC (Figure S2). The TT samples were not included in the SEC analysis as they were viscous at room temperature, which resulted in clogging of the HPLC system. As SDS-PAGE resolves most proteins at proper conditions, the TT samples were therefore subjected to SDS-PAGE (Figure S3). 

Of the four proteases exhibiting the highest TT/CM ratio (i.e., Endocut-02 and Endocut 03), Endocut-02 was chosen for further studies based on the SEC chromatograms of the MDCR hydrolysis. This is because Endocut-02 showed the highest relative share of high-molecular-weight fractions in the SEC chromatograms, strongly indicating the presence of larger collagen-derived fragments (Figure S2). The retention times of the calibration standards can be found in Table S2. This was further supported by the SDS-PAGE separation, with Endocut-02 being one of the proteases yielding the highest amount of high molecular weight (Mw) peptides relative to low Mw peptides, together with Protamex and Alcalase (Figure S3). Endocut-02 is an alkaline subtilisin type protease belonging to the serine proteases (EC no. 3.4.21.62), showing broad-spectrum specificity with a preference for large uncharged residues in P1 [17]. It is produced by controlled fermentation of *Bacillus licheniformis*.

Of the four proteases exhibiting the lowest TT/CM ratio (i.e., Bromelain, FoodPro 51, and Corolase 2TS), Bromelain extracted from the stem of a pineapple (EC 3.4.22.32) was chosen due to its well-known non-specific action on proteins and its activity towards collagen [18]. Bromelain was also the protease exhibiting the highest amount of protein bands below 37 kDa, relative to the number of protein bands with a Mw above 37 kDa, after SDS-PAGE separation of all hydrolysates (Figure S3). Bromelain has been used, amongst all, to increase collagen yields in surimi processing and has been recognized as a suitable protease for the production of gelatin and collagen-derived hydrolysates [8,19].

Stem Bromelain has a preference towards cleaving peptide chains in between two Arg residues, especially in small peptides, but also cleaves substrates with a Arg or Lys in P1 position [20,21]. This difference, i.e., proteases being more or less sensitive to different substrates was also observed by, e.g., Fu et al. [22]. In their study, Fu et al. compared yield after hydrolysis of porcine plasma and ground bovine meat using 10 different proteases at optimized conditions for each protease. Alcalase was shown to have relatively poor selectivity, while Bromelain was more sensitive to the substrate. Furthermore, in the Fu study, Flavourzyme was more sensitive to the substrates used than what is shown here. This only stresses the importance of not assuming that activity differences seen between one set of substrates will be the same regardless of the substrate type, but rather that each set of targeted substrates might have a different set of optimal proteases.

### 2.2. Proteolysis of Poultry Substrates Using Bromelain and Endocut-02

The selected proteases, i.e., Bromelain and Endocut-02, were used in EPH of single and mixed materials (CM, TCT, TCT+CM, MDCR) using a lab-scale stirred reactor. The TCT consisted of a mixture of turkey collagen-rich tissues, as opposed to the TT material used in Section 2.1. The upscaling of the reaction was done partly to investigate if the small-scale screening results were comparable to reactions in this setting, but also to investigate in greater detail the differences in activity during hydrolysis and in product characteristics. The composition of the raw materials used in this part of the study is found in Table 2. The protein, fat, and ash content of CM and MDCR correspond to an earlier study of poultry by-products of Norwegian origin [23]. Hydroxyproline (Hyp) is found almost exclusively in collagen, and the amount of Hyp is frequently used to estimate collagen contents in tissues [24]. Thus, Hyp concentrations of all materials were measured and have been converted to estimated collagen values in Table 2. As expected, the highest concentration was found in the TCT raw material, the lowest in CM, and an intermediate value was found in the MDCR material.

The resulting protein yields after 60 min hydrolysis are presented in Table 3. The upscaling of the hydrolysis results in generally higher yields and lower standard derivation (SD) between duplicate rounds as compared to small-scale values. This stresses the importance of treating yield results from screening in small-scale hydrolysis set-ups as indicative results. Furthermore, the use of water (pH ca 6.2) in hydrolysis instead of sodium phosphate buffer at pH 7.0 for both proteases resulted in a decent protein recovery considering that the temperature used is in the low range of the temperature curve for both proteases. Bromelain generally resulted in higher protein recovery than Endocut-02 in hydrolysis of all substrates. This shows that relatively small changes in pH can result in changes to the activity of the proteases used.

**Figure 1:**
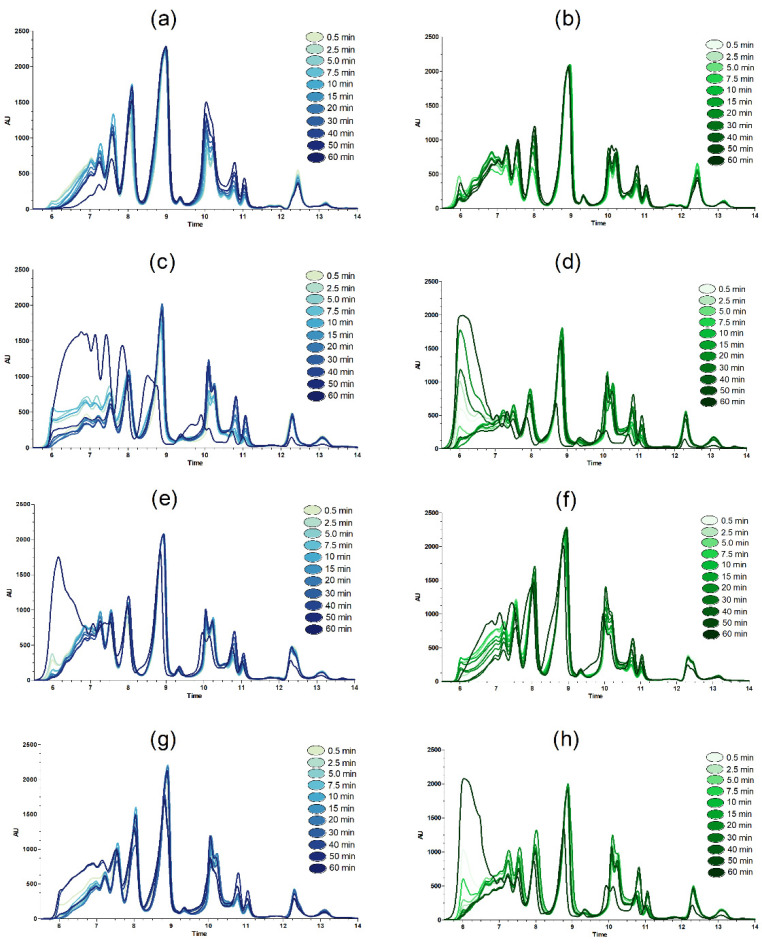
SEC chromatograms from time-series sampling during lab-scale hydrolysis (average of duplicate measurements) with the following materials and proteases: (**a**) CM, Bromelain, (**b**), CM, Endocut-02, (**c**), TCT, Bromelain, (**d**) TCT, Endocut-02, (**e**) TCT+CM, Bromelain, (**f**) TCT+CM, Endocut-02, (**g**) MDCR, Bromelain, (**h**) MDCR, Endocut-02. The time series colors are given in the figure.

The Mw distribution of peptides from all of the time series is presented in the SEC chromatograms of Figure 1. The SEC method used is optimized for maximum resolution in the low Mw range, and the retention times of the calibration standards can be found in Table S2. The chromatograms show clear differences in protein breakdown patterns related to both proteases and substrates involved. From the SEC chromatograms resulting from CM hydrolysis, there seems to be a tendency for Endocut-02 (Figure 1b) to produce peptides with a higher Mw than Bromelain (Figure 1a). This is indicated by chromatograms at later hydrolysis time points, showing larger peaks in the 6–8 min range for Endocut-02, and the higher amounts of small peptides (≤ 600 Da) seen in the Bromelain hydrolysates eluting between 10–11.5 min. This tendency can also be seen in the TCT+CM substrate (Figure 1e,f), containing a 50/50 mix of chicken meat and turkey collagen tissue. For all substrates containing high amounts of collagen proteins, i.e., TCT (Figure 1c,d), TCT+CM (Figure 1e,f) and MDCR (Figure 1g,h), large changes in the Mw distribution were evident in the 60 min samples, specifically in the region with the highest Mw compounds (i.e., 5.5–8 min). To stress the importance of this, notice that the only difference between the 50 min samples and the final 60 min samples are the conditions for terminating the reaction. For the 50 min samples, samples were devoid of sediment at inactivation, while for the 60 min end hydrolysates, all remaining sediment fractions were present at inactivation.

The protein concentrations of all dried hydrolysates are presented in Table 4, showing that Bromelain generally resulted in higher protein concentrations than Endocut-02. However, for the 60 min TCT hydrolysates, calculated protein contents of 96.9, or even 92.8 g/100 g are unlikely high. The authors believe this to be an effect of high levels of collagen within these samples. Collagen contains higher nitrogen levels than muscle proteins, over 18% and 16%, respectively. The difference in nitrogen levels is a result of differences in the amino acid composition between the two protein classes, with collagen containing more of low Mw amino acids [26]. Consequently, the nitrogen-to-protein ratio will vary as a function of raw material when the collagen content varies. Due to the high raw material complexity in the current study, involving mixes of muscle and tendons, the muscle-derived conversion factor of 6.25 has been used for all raw materials [27].

**Figure 2:**
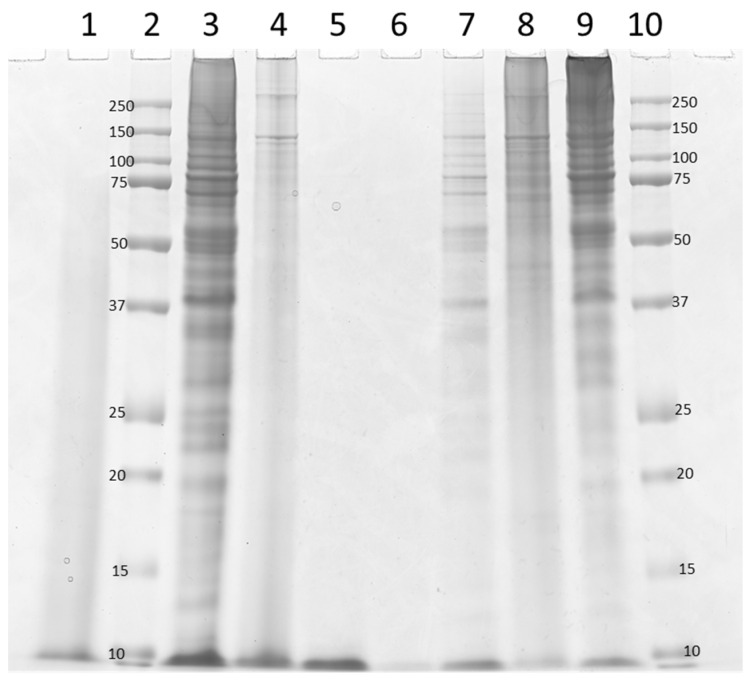
Hydrolysates resulting from Bromelain (-B) and Endocut-02 (-E) hydrolysis on four different raw materials, and Mw marker (sizes given in the figure), run on a 12% SDS-PAGE gel. From left to right: (1) TCT+CM-B, (2) Mw marker, (3) TCT+CM-E, (4) MDCR-B, (5) CM-E, (6) CM-B, (7) MDCR-E, (8) TCT-B, (9) TCT-E, (10) Mw marker.

Table 4 also supports the findings from SEC, i.e., that there are indications of differences in hydrolysates stemming from CM as compared to the collagen-rich materials. This can be seen in the protein content difference of dried samples taken at t = 50 min and the final 60 min hydrolysates, which were larger for the more collagen-rich raw materials TCT and MDCR than for CM, with the mixed TCT+CM material showing intermediate values. The results indicate that something is released at inactivation from the collagen-rich materials. As the SEC method employed was optimized for high resolution in the low Mw range, further insight into the background for differences in peptide size distribution and the proteins of the high-Mw fractions of the hydrolysates was provided by SDS-PAGE separation using gels with a high resolution in the high-Mw range (Figure 2).

In Figure 2, lanes 5 and 6, separation of hydrolysates from CM for both proteases is shown. The absence of protein bands indicates that the peptides in the CM hydrolysates were digested to peptides less than 10 kDa in size, corresponding to approximately 7 min in the SEC chromatograms (Figure 1). In Figure 2, lanes 8 and 9, hydrolysates resulting from the digestion of the collagen-enriched TCT material are shown. These lanes show the presence of protein bands of all sizes, with an emphasis on proteins with Mw above 50 kDa, especially in the Endocut-02 hydrolysates (lane 9). The presence of protein bands with Mw above 100 kDa indicates the presence of undigested collagens. Earlier studies have shown that reduced α_1_- and α_2_-chains (monomeric form) from turkey collagen I migrated to approximately 110 and 120 kDa, while the β- and γ-bands (di- and trimeric forms, respectively) migrate to a Mw above 240 kDa [28,29,30,31]. Moreover, in an SDS-PAGE separating pepsin-solubilized turkey tendon collagen, Grønlien et al. showed the presence of several forms of α collagen as well as the presence of β and γ forms of collagen molecules [32]. In more detail, on the Grønlien gel, α-chains from both collagen I and III were present in between the Mw standard bands corresponding to 82 and 115 kDa. From low to high Mw, these were α_2_(I), α_1_(I), and α_1_(III). The Mw of the bands in Figure 2 were corresponding to several types of β-chains, which could be approximated to around 200 kD, and the size of several present γ-chains even higher in Mw. These Mw’s were also in accordance with those described by Du et al. after running SDS-PAGE on gelatin extracted from chicken and turkey heads [33].

The differences between the proteases seen after the hydrolysis of TCT were also seen resulting from TCT+CM hydrolysis (Bromelain, lane 1; Endocut-02, lane 3). While Bromelain results in a hydrolysate without evident undigested collagens or defined bands, as also indicated in the corresponding SEC chromatograms (Figure 1e), the Endocut-02 hydrolysate contains a fair number of bands corresponding to the pattern seen in the TCT hydrolysate (lane 9). In the digestion of MDCR (lanes 4 and 7), however, the picture is slightly different. Here, results indicate the presence of α-, β- and γ-bands after hydrolysis of both proteases, but as earlier, in a higher number in the Endocut-02 hydrolysate (lane 7) than the Bromelain hydrolysate (lane 4).

In conclusion, the protein increase seen in collagen-rich samples in Table 4 was indicated by SEC and SDS-PAGE results to stem from solubilized, partly digested, and degraded collagen, i.e., gelatin. More specifically, α- and γ-chains were liberated after heat inactivation and hydrolysis with both proteases. This is supported by the fact that especially the TCT hydrolysates were highly viscous at room temperature (data not shown). Additionally, results indicate the presence of the dimer form of collagens, i.e., β-chains, resulting from Endocut-02 digestion of TT (lane 9). Lastly, it should be stressed that not only amount, but also the exact band positions in SDS-PAGE gels differ slightly between hydrolysates, meaning that differences are present in both the amount and absolute size of the solubilized gelatin molecules.

### 2.3. Combination of Proteases in EPH of MDCR

To investigate if there were beneficial effects on protein yield and effects on product composition by using proteases exhibiting differences in selectivity, hydrolysis using a combination of the two proteases were compared with hydrolysates resulting from the use of either Endocut-02 and Bromelain separately. Because of the low yield in Section 2.2, increased enzyme over substrate (E/S) ratios was used for these experiments. Three different modes of a combination of the two proteases were used, one where Bromelain and Endocut-02 were used together for the whole hydrolysis time (BE), and two where Bromelain was added 30 min before Endocut-02, and vice versa (B+E, and E+B). The protein yield as well as dry matter protein concentrations resulting from duplicate hydrolysate runs are presented in Table 5.

Comparing single-protease hydrolysis reactions, using a slightly higher enzyme concentration, Endocut-02 showed slightly higher efficiency in hydrolyzing MDCR than Bromelain. This relative difference corresponds to results from Section 2.1 where dry matter yield was evaluated. Furthermore, the Endocut-02 yield increased by 35% relative to conditions used in Section 2.2 when increasing the E/S ratio, while the Bromelain activity was basically the same. This verifies that optimization trials, including E/S ratio optimization, should always be considered in a cost/benefit perspective in process optimization. Comparing to single protease hydrolysis, a close to 15% increase in protein yield was observed when EPH was performed using a combination of proteases. Additionally, studying the effects of different ways to combine proteases, i.e., E+B, B+E or BE, results showed that there were no significant differences in yield. This was rather surprising, considering that during B+E and E+B hydrolysis, the full protease concentration was only reached after 30 min as opposed to BE where both proteases were present during the whole hydrolysis.

Table 5 also shows very high calculated protein concentrations in the final hydrolysates. As seen and argued in association with Table 4, the very high protein concentrations are likely due to the presence of large quantities of proteins and peptides from collagen, with a relatively higher nitrogen content than meat-derived proteins, in the final product. The protein concentration of the 60 min samples (where sediment was removed before inactivation) was lower than in the end product hydrolysates (where sediment was present at inactivation), especially for the single protease reactions. In addition, using the higher E/S ratio, this effect was even more pronounced than seen in Section 2.2, indicating that use of a longer time or even higher protease concentrations could result in higher yields. 

For combined hydrolysis, the difference in protein content between the 60 min sample (without sediment at inactivation) and the end product hydrolysates (with sediment at inactivation) were lower than for the single protease reactions, irrespective of the combination of proteases. The fact that the difference still existed indicates that collagen-derived proteins/peptides were still liberated from the sediment when applying the high temperatures during inactivation, even though the total yields were now higher. In a study by Offengenden et al., single and combined protease hydrolysis were performed on collagen extracted from chicken meat [14]. In this study, four proteases were used separately in a 2 h hydrolysis at 2% (*w*/*w*) concentration using the optimum pH for each protease. The resulting protein yields were between 35 to 61%. A second round of hydrolysis where later performed, using four combinations of two of the earlier used proteases (2% (*w*/*w*), sequential reaction: 2 × 2 h at optimum pH for each protease). This resulted in an increase in protein yield of 82–90%. Considering the use of a much longer hydrolysis time, higher protease concentrations as well as optimized pH conditions, the current protein yields of ca 70% in hydrolysis of MDCR at lower temperature could be considered promising.

**Figure 3:**
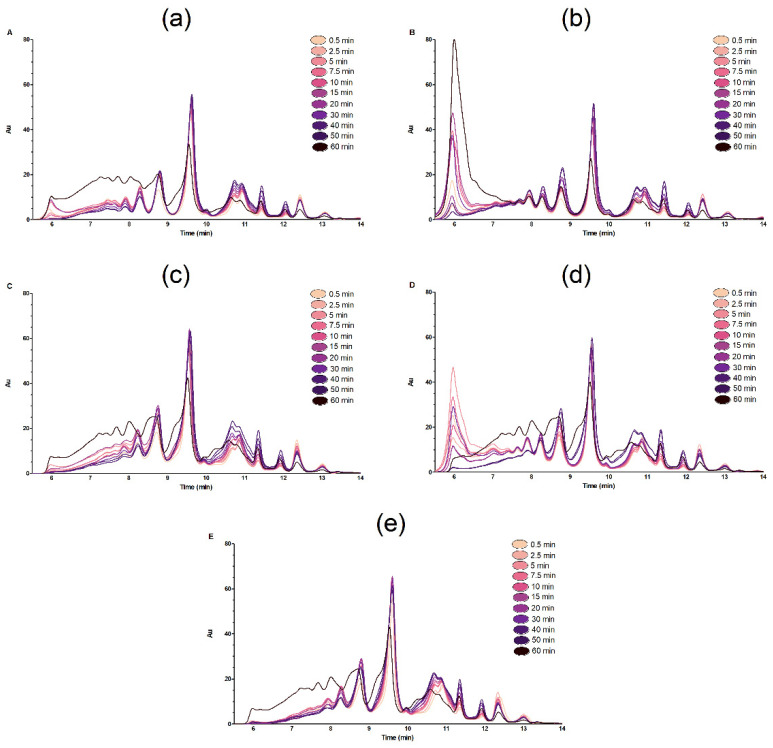
The resulting time SEC chromatograms from time series samples taken from hydrolysis of MDCR using single or mixed enzymes. The proteases used in the (**a**–**e**) time series insets were: (**a**) Bromelain, (**b**) Endocut-02, (**c**) B+E, (**d**) E+D, and lastly, (**e**) BE. The time series colors are given in the figure.

**Figure 4:**
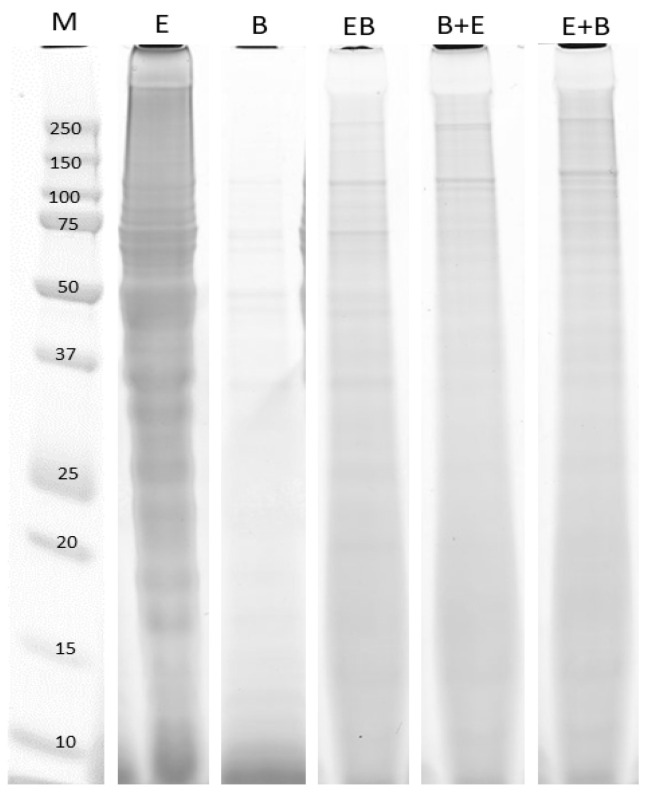
The resulting SDS-PAGE gels of hydrolysates from mixed and single proteases after inactivation. The figure shows a collage of lanes cut from one gel. In the left lane, the Mw marker (M) with the size of the Mw standard proteins given (kDa).

The hydrolysates were further subjected to SEC and SDS-PAGE, seen in Figure 3 and Figure 4, respectively, to investigate in greater details the effects of combining the proteases.

Again, as seen in Figure 1, the resulting Mw distribution of peptides from single protease hydrolysis (Figure 3a,b) show the largest differences in the high Mw region (5.5–7.5 min), both during the reaction but also in the 60 min product hydrolysates. In EPH using the E+B and B+E combinations, there is a “signature proteolysis pattern” in the high Mw region, recognizable from the pattern seen from the single protease during the first 30 min. Thereafter, the changes happening between 30–60 min act to equalize the differences in Mw size distribution, resulting in chromograms for the 60 min final hydrolysates that were very similar for E+B and B+E. This was also valid for the BE chromatogram where the two proteases were added together at t = 0 (Figure 3, panel E). The fact that the final 60 min hydrolysate chromatograms resulting from all mixed proteases were similar corroborates the findings of Table 5, showing that when performing mixed protease hydrolysis, the final hydrolysates will achieve same properties irrespective of the way proteases were mixed. This was further validated by running the hydrolysates on an SDS-PAGE gel (Figure 4). The SDS-PAGE results showed that the pattern resulting from proteins/peptides separated in the lanes containing BE, E+B and B+E final hydrolysates were very similar.

Interestingly, the difference in hydrolysis conditions with higher relative concentrations of proteases, compared to hydrolysates shown in Figure 2, has resulted in differences in visible protein bands in the lanes for single protease MDCR hydrolysates in Figure 4. In the latter, there is a significant decrease of high Mw protein bands in the 150–250 kDa region, compared to the MDCR Endocut and Bromelain lanes in Figure 2. For Endocut, the new hydrolysis conditions have resulted in a smear in the 150–250 kDa region, while for Bromelain, no visible protein bands were present. This was also valid for the 100–150 kDa range. Building on the discussion in association to Figure 2, this means that use of higher protease concentrations, formation of mono-, di-, and trimeric forms of collagen-derived molecules has been inhibited. For Bromelain, it can be postulated that higher concentrations of protease could facilitate the formation of more of the smaller peptides, even though the overall protein yield was not increased. However, for the Endocut-02 protease, it seems like the increased protease concentration serves to increases the amount of non-specific cuts in the high-molecular-weight region, evident by the lack of clear protein bands. Further studies using an additional set of experiments at other hydrolysis conditions will be needed to shed more insight into this matter.

### 2.4. General Discussion and Industrial Relevance

In this study, the effects of single and combined protease hydrolysis for the extraction of meat-based and collagen tissue-based proteins from poultry by-products has been investigated. The focus was set on the extraction of proteins from collagen as this is harder to solubilize by enzymatic means than meat proteins. Two proteases showing vastly different properties in the hydrolysates from the initial screening were selected for further studies. Results show that they indeed work very differently on these materials also on a larger scale. Our hypothesis for explaining the differences seen in Bromelain and Endocut-02 products is based on their respective selectivity differences. As stated earlier, stem Bromelain prefers Arg and Lys residues in P1, while the subtilisin type Endocut-02 prefers hydrophobic amino acids [17,20,21].

From a collagen functional perspective, Arg is an important amino acid for stabilizing the triple helix structure, and Lys is important for telopeptide cross-linking [34]. Even though the repetitive amino acid pattern in the fiber region of mature collagen might act to shield some of the cleavage sites, our hypothesis is that the cleavage pattern inferred by Bromelain selectivity might act to weaken the structure both within the repetitive fiber structure and in the telomers, thus allowing for solubilization of relatively small peptides throughout hydrolysis and at inactivation. This is corroborated by the results from the current study, which shows that Bromelain hydrolysates mainly contain a fair number of small peptides, also in EPH of collagen-rich materials, although some larger segments were also solubilized. On the other hand, for Endocut-02, large hydrophobic amino acids are known to be virtually non-existent in collagen molecules. If cutting mostly out of the repetitive core fiber region, i.e., in the telopeptide regions, the fiber structure should be left mostly untouched. If correct, it is only when the partly cleaved collagen structure is heated to 95 °C that enough energy is added, facilitating a break the stability of the triple helix collagen molecules. This breakdown of the collagen network should be further facilitated by the fact that telopeptide region is important for fiber stability [34]. 

Indeed, evident by both SDS-PAGE and SEC results, relatively large collagen-based proteins, i.e., gelatin, were solubilized after inactivation of Endocut-02. The SDS-PAGE gel in Figure 2 showed the presence of a relatively high number of intact α-, β- and γ-bands as a result of Endocut-02 hydrolysis. The SEC chromatograms also showed the presence of more of the large proteins in the Endocut-02 hydrolysates than in the Bromelain hydrolysates. It is well known that gelatin is liberated from samples by heating, and that the effect can be emphasized when the material has been pre-treated with proteases [9]. Yet, heat-induced solubilization of collagens versus myofibril proteins following standard enzymatic protein hydrolysis reactions of complex side-stream materials have not been extensively studied. There are examples where Alcalase, Bromelain, Papain, among other proteases have been used to facilitate collagen digestion and extraction, but these studies do generally not include how the collagen yields and digestion levels evolve during EPH reactions prior to the inactivation step [35,36]. 

Based on the results of the current study, where it was indicated that a sizable fraction of the collagen was solubilized with different Mw distributions at the inactivation step, it was realized that a better understanding of the process could have implications in industrial EPH process development. To get to that point, further studies will be needed to elucidate the selectivity difference and its potential effects of solubilizing collagen, and to verify if the presence of intact fiber molecules is indeed an effect of heating the sediment fraction during inactivation. However, the dependence of choice of protease on both the amount and size of collagen proteins being liberated during heat treatment was, to the authors’ knowledge, highlighted here for the first time.

A consequence of larger collagen proteins in the form of gelatin being solubilized from the sediment during inactivation is that this feature can be utilized in industrial processing to retrieve fractions enriched in myofibrillar and collagen peptides on the one hand, and gelatin as a separate product on the other. This could potentially all be achieved in one process. The rationale would be that collagen peptides have vastly different properties than collagen proteins in, e.g., food, and the tailor-made products could be sold in different markets [37]. One possible solution to achieve this is to perform a decanter-based separation of sediment and hydrolysate before inactivation, adding new water to the sediment fraction, and thereafter heat-inactivating the hydrolysate and the sediment/hydrolysate fractions separately to allow for the collagen proteins to be solubilized from the sediment fraction. Another solution would be to perform a normal heat inactivation of the whole hydrolysis mixture, and use a decanter to separate hydrolysate from sediment, where after an ultra/micro-filtration under optimized conditions could serve to separate the larger gelatin molecules from the smaller completely solubilized peptides. Hence, to the authors’ knowledge, this study shows for the first time shows that if selecting the appropriate protease, a process based on a one-step EPH hydrolysis of MDCR can be developed that results in both gelatin and collagen/myofibrillar peptides. Further possibilities in tailor-making hydrolysates can also be derived from the results of combining proteases. Although the obtained final products have very similar properties, the time series of Figure 3 clearly reveal that the mechanisms leading to the final products are different. Thus, combining the proposed one-step EPH hydrolysis alternatives discussed above with a rational addition of proteases at different timepoints is likely to provide additional possibilities in producing protein and peptide products with highly tailor-made properties from MDCR. Research will be needed to evaluate the potential of such alternatives.

The result from the normalization of the protease activity can be used for calculating the hydrolysis cost per unit product converted. This has been done in a comparative study of cost-efficiency between different proteases using the casein assay, where Aspevik et al. concluded that the cost per casein activity differed 2.7 times between the highest and lowest cost per casein unit out of the five proteases examined [3]. Much can be saved from a production standpoint by performing such studies, with the additional benefit of obtaining protease values using one assay only, as opposed to the various proteases assays used by the commercial protease vendors to report protease activity. However, the resulting activity will be different using other temperature and pH conditions, as well as if choosing a different substrate. As no industrial standard is implemented, this means researchers and companies must perform separate cost-efficiency studies. If aiming at further insight into the cost-efficiency of single raw materials, e.g., collagen, studies should arguably be performed using synthetic substrates with a higher resemblance to the raw material, for example, azocoll. The hydrolysis conditions used in the current study, i.e., a hydrolysis temperature of 40 °C, are lower than standard industrial hydrolysis temperatures used. Although savings clearly can be made by using lower temperatures for food safety reasons and from the simple fact of fat being more soluble at 50 °C, to the authors’ knowledge, few industries run hydrolysis reactions at these low temperatures. However, as the hydrolysate is being pasteurized at inactivation, the study presents industrially relevant results for EPH processes with short hydrolysis times (less than 1 h), showing that high yields can be achieved using less energy which not only saves costs, but also contributes to a more sustainable industry. There is also a potential for cost-reduction by use of water instead of acidic/alkaline pH normally used for collagen extraction. The use of no pH-adjusting chemicals not only saves the chemical costs, but also leads to a reduced amount of unit operations involved in downstream processing for salt reduction. However, the optimal compromise between processing conditions, including choice of protease and the concentration used, will be dependent on existing infrastructure and production investments (CAPEX) and operational costs (OPEX) of each EPH processing plant [38].

## 3. Materials and Methods

### 3.1. Materials and By-Products

CM, TT, TCT, and MDCR were supplied from a Norwegian slaughterhouse (Nortura, Hærland, Norway). However, for the Section 2.1 activity screening, both CM and chicken thighs for the CB material were purchased at a local food store (Ås, Norway). All the raw materials were frozen at −20 °C on the day of arrival. For the Section 2.1 hydrolysis, CM was homogenized using a blender (BL-1200, Wilfa, Norway). To prepare the minced CB material, chicken thighs were thawed, after which meat was removed from the bones, first by knife but later by scrubbing the bones under lukewarm water. The femur and tibia were separated from the knee and metatarsus by-products. Using a pruning shear, the femur and tibia bones were cut into approximately 1-cm pieces and put into the −40 °C freezer ON. After pouring liquid N_2_ over the frozen pieces of bone, they were ground by adding small aliquots (approximately 15 pieces per round) to a stainless-steel food blender, using 15 pulses per round.

The TT material consisted of manually removed turkey Achilles tendons (at the Nortura slaughterhouse), that were further manually scraped on lab to remove all remains of meat using a knife. The clean Achilles tendons were cut into 0.5 × 0.5 cm pieces and frozen to −40 °C until the day of hydrolysis. CM for Section 2.2 and all MDCR raw materials was thawed over night at 4 °C before being minced using a 1-cm hole plate in a Seydelmann SE130 meat grinder (Seydelmann, Stuttgart, Germany). Preparation of the TCT materials involved cutting frozen blocks of mixed turkey collagen by-products to approximately 3 × 5 cm pieces using a meat saw, followed by grinding using a table-top meat bowl cutter. The TCT+CM material was prepared by mixing CM and TCT in a 50/50 ratio. All Section 2.2 materials were vacuum-packed in individual packages and stored at −20 °C until hydrolysis. The chemicals used in analysis where origin is not specified were purchased from Sigma-Aldrich (St. Louis, MO, USA).

### 3.2. Activity Determination Using the Azo-Casein Assay

A modified version of the Megazyme endo-protease azo-casein assay description (S-AZCAS 12/07, Megazyme, Bray, Ireland) was used. Substrates were prepared according to instructions, and each protease was solubilized in the recommended buffers, meaning that for all proteases except Bromelain, Promod 144L-100TU, Promod 950L and Veron L, the preferred standard buffer consisted of sodium phosphate (0.1 M, pH 7.0). For the four mentioned proteases, the standard buffer was supplemented with cysteine and EDTA according to the assay description. For the assay, 2 mL Eppendorf tubes with substrate or protease solutions were prepared and placed in a thermomixer (TermoMixer F1.5 from Eppendorf, Germany) at 42 °C, 500 rpm. The use of 42 °C was shown to be needed to guarantee that the liquid phase obtained a temperature of 40 °C after pre-heating. After 20 min, at t = 0, 200 µL of the respective protease solution was added to 200 µL of substrate solution. After protease addition, each tube was vortexed for 2 s and incubated for precisely 10 min. The reaction was terminated by addition of 1.2 mL 5% (*w*/*v*) trichloroacetic acid (TCA), followed by vigorous stirring for 3 s using a vortex mixer. For the blank sample, immediately after the addition of TCA, the enzyme solution with the lowest concentration was added to the TCA/substrate mixture. Five min after TCA addition, all reaction tubes were centrifuged (5200 rpm, 10 min in a table-top centrifuge (MICRO-STAR 17R from VWR, Radnor, PA, USA). The supernatant consisting of hydrolyzed azo-casein was transferred to plastic disposable cuvettes, and the absorbance was read against the reaction blank at 440 nm using a spectrophotometer (Ultrospec 3000, Pharmasia Biotech, Cambridge, UK). Using the linear regression function in GraphPad Prism 8 (GraphPad Software, San Diego, CA, USA), a fit using a linear function (𝑦 = kx + m) was performed for the spectroscopy data for each protease, and the dilution for each protease at y = 1 was calculated. The dilution at y = 1 for each protease formed the basis of the amount of protease used in the following steps.

### 3.3. Screening of 18 Proteases on Different Poultry Raw Materials

The names, vendors as well as properties of the 18 proteases are listed in Table 6.

All raw materials were prepared before starting the experiment, weighing 2 g (between 2.000–2.050 g) of raw material into a 10 mL tube (79 × 16 mm, Sarstedt, Germany). The weights of the sample and the empty tube were noted, after which all samples were stored in a −40 °C freezer before use. On the day of hydrolysis, tubes were thawed in cold water. An amount of 7.5 mL of the sample buffer, 0.01 M sodium phosphate, was added to each tube, whereafter the samples were pre-heated for 10 min in a water bath at 45 °C to achieve a sample temperature of 40 °C. At t = 0, 1 mL of an individual concentration, as explained in Section 2.1, of each protease (diluted 1:10 in sample buffer) was added to each sample, except for the background samples. 

After addition, the samples were place in an end-over-end mixer which was placed in a heating cabinet (TS8136, Termaks, Norway) for incubation at 42 °C. After hydrolysis of samples and background reactions were finished, samples were placed in a water bath at 95 °C for 20 min for inactivation. Inactivated samples were vacuum filtrated using a Büchner flask with a glass funnel and a 597 Whatman filter paper to separate sediment from hydrolysate. The filter paper and funnel were pre-heated with hot water before filtration to enable the full separation of gelatin-rich collagen samples. After filtration, the filter papers, including residuals, were dried until completely dry in the heating cabinet at 50 °C, in addition to the empty tubes after filtration in case of remaining samples. The dried filters and tubes were weighted for the calculation of weight yields.

### 3.4. Lab-Scale Enzymatic Hydrolysis

Lab-scale proteolysis reactions were performed according to Wubshet et al. with modifications as described below [39]. EPH was first performed on CM, TCT, TCT+CM or MDCR using either Bromelain or Endocut-02. In these trials, 333 g of raw material were mixed with 667 g of grade 3 Milli-Q water using 1149 µL of Endocut-02 or 1517 mg of Bromelain. In the second round of proteolysis reactions, mixtures of Bromelain (1426 mg) and Endocut-02 (1078 µg) or single enzymes (Bromelain 2852 mg, Endocut-02 2156 µL) were used to hydrolyze MDCR. In these proteolysis reactions, 250 g of raw material was mixed with 500 mL of Milli-Q water. Hydrolysis was performed under stirring (300 rpm) in a preheated (40 °C) jacketed reaction vessel. The reactions were started at t = 0 by adding the enzymes, dissolved in 10 mL of water for 30 min prior to this. The total reaction time was 60 min. During the hydrolysis, aliquots of approximately 10–15 mL were collected at 12 time points (i.e., 0.5, 2.5, 5, 7.5, 10, 15, 20, 30, 40, 50 and 60 min, respectively). Background reactions with raw materials only, using the same conditions as when proteases were added, were also performed. All reactions were performed in duplicates. All phases were recovered from the respective containers and weighted for future reference. The hydrolysates were aliquoted in 250 mL plastic packages with lids and stored frozen at −40 °C until lyophilized (CHRIST 1–16 LSCplus, Germany). For SEC and FTIR measurements, an aliquot of the water fraction after proteolysis and background reactions were filtered through a Millex-HV PVDF 0.45 μm 33 mm filter (Millipore, Billerica, MA, USA). SDs of duplicate values were calculated by the STDEV.S function in Excel (Microsoft, Redmond, Washington, USA), involving Bessel’s Correction.

### 3.5. Chemical Composition and Hyp Content in Hydrolysates

The TCT, CM and MDCR raw materials in Section 2.2 were sent to a commercial analytical lab (ALS Global, Oslo, Norway) for analysis of protein, fat, and ash composition and Hyp content. Shortly, ALS global defined the following methods: ash by gravimetric method (BS 4401 Part 1, 1998, Commission Regulation (EC) 152/2009 (measurement uncertainty (MU) 6.5%); protein content by the Dumas method (MU 1.8%) and a protein conversion factor of 6.25; fat content by pulsing NMR (MU 6.5%); Hyp content by spectrophotometry (EU standard BS 4401–11:1995) (MU 17.4%). SDs of duplicate values were calculated by the STDEV.S function in Excel (Microsoft, Washington, USA), involving Bessel’s Correction.

### 3.6. Nitrogen Recovery and Protein Content

The nitrogen content for all the hydrolysis samples and raw materials was measured by the Dumas combustion analysis. While the nitrogen content of the raw materials was measured by ALS Global, Oslo, Norway, the lyophilized hydrolysates were analyzed using a Vario EL cube instrument (Elementar, Langenselbold, Germany) and sulfanilamide as a correction standard. For analysis, about 5 mg samples were packed in tin foil as described by Rieder et al. [40]. A nitrogen-to-protein conversion factor of 6.25 was used to estimate the protein content.

### 3.7. SEC

The SEC analyses were carried out as described by Wubshet et al. [38]. Calibration standards with molecular masses ranging from 204–66,463 g/mol were prepared as 2 mg/mL aqueous solutions. The standards used for calibration are presented in Table S2. The samples were prepared as 25 mg/mL solutions in the mobile phase and filtered using a Millex syringe filter with a PVDF membrane (pore size 0.45 μm, Merck Millipore, Burlington, MA, USA). The SEC separations were performed with either an Agilent 1100 series instrument (Agilent Technologies, Santa Clara, CA, USA) or a Dionex Ultimate 3000 instrument (Thermo Scientific, Waltham, MA, USA) fitted with a SecurityGuard HPLC guard cartridge system and a BioSep SEC-S2000 column (300 mm long with an inner diameter of 7.8 mm, Phenomenex, Torrance, CA, USA). The mobile phase consisted of 30% acetonitrile and 0.05% trifluoroacetic acid in Milli-Q water (*v/v*). An injection volume of 10 μL was used for both the calibration standards and the samples. The chromatographic runs were controlled from either OpenLAB CDS ChemStation Rev. C. 01.07 (Agilent Technologies) or the Chromeleon software version 7.2 SR 4 (Thermo Schientific, Walham, MA, USA). From the chromatographic runs of both the standards and hydrolysates, a UV trace of 214 nm was monitored. The chromatographic data were processed using PSS WinGPC UniChrom V 8.33 (Polymer Standards Service, Mainz, Germany).

### 3.8. SDS-PAGE

Samples from the small-scale raw material screening (Section 2.1) were heated 10 min at 80 °C. 10 µL of each sample were mixed with 10 µL Pierce LDS Sample Loading Buffer (Thermo Fisher Scientific, Waltham, MA, USA) and 4 µL 1 M dithiothreitol (DTT). The samples were heated again 10 min at 80 °C. 10 µL of each sample was loaded in the well. Proteins were separated at 200 V using 12% Bis-Tris Nu-PAGE gel and MOPS running buffer (Thermo Fisher Scientific). Precision Plus Protein Dual Xtra Standard was used as a protein marker (BioRad, Hercules, CA, USA). The gels were stained/destained following the Simply Blue SafeStain protocol (Invitrogen, Carlsbad, CA, USA). After freeze-drying, samples from the lab-scale hydrolysis (Section 2.2 and Section 2.3) were ground using a mortar, and 25 mg was transferred to a 1.5 mL Eppendorf tube and added 500 µL Milli-Q water to a final concentration of 50 mg/mL. Samples were incubated for 30 min at 50 °C, shaking at 800 rpm. Tubes were centrifugated 10 min at 3200 rpm at room temperature and the supernatant transferred to new clean tubes. An amount of 20 µL of each sample was mixed with 20 µL of SDS-loading buffer (0.125 M Tris, 4% SDS, 20% glycerol, 0.2 M DTT and 0.04% bromophenol blue). Samples were heat-treated 15 min at 50 °C shaking at 400 rpm. Ten µL of each sample was loaded in the well. Proteins were separated as described above.

## 4. Conclusions

After a substrate-based screening of 18 proteases on four different poultry-based by-products, stem Bromelain and Endocut-02 were selected due to the greatest apparent activity differences. Further studies showed that choice of protease and ultimately selectivity-related differences in cleavage of the collagen structure resulted in differences in both amount and size of collagen proteins being liberated during heat treatment. Endocut-02 hydrolysis resulted in a hydrolysate rich in both myofibrillar and collagen peptides, as well as in gelatin. This indicates that a one-step process can be developed for the valorization of MDCR, resulting in two collagen-rich industrially relevant products with vastly different physicochemical properties. Further, when combining the two proteases in three different combinations for EPH of MDCR, products with very similar yield, SEC and SDS-PAGE profiles were achieved. This shows that the mode of combination has little impact on final product properties. All combinations of proteases resulted in an approximately 15% protein yield increase as compared to single protease EPH. This suggests that the use of a combination of proteases could be a viable method to achieve better total utilization and ultimately better valorization of complex by-products.

## Figures and Tables

**Table 1:** Weight yield (g/100 g) after hydrolysis of turkey tendons (TT), chicken bones (CB), chicken meat (CM) and mechanically deboned chicken residues (MDCR) using protease concentrations given in Table S1 for one hour (single hydrolysis). In the right column, the resulting ratios of weight yields from TT over CM.

	Weight Yield (g/100 g)				
	TT	CB	CM	MDCR	Ratio TT/CM (%)
**Alcalase**	43	11	50	41	86
**Bromelain**	46	28	55	37	83
**Corolase 2TS**	63	28	74	42	85
**Corolase 7090**	59	22	52	29	114
**Endocut-01**	50	32	49	36	103
**Endocut-02**	45	35	33	37	136
**Endocut-03**	51	20	41	29	124
**Flavourzyme**	36	13	38	25	94
**FoodPro 30L**	45	23	44	35	102
**FoodPro 51 FP**	48	17	64	31	74
**FoodPro PNL**	50	16	45	37	109
**Protamex**	63	7	56	34	114
**Promod 144GL**	48	17	50	44	96
**Promod P950 L**	37	17	42	31	88
**Tail-10**	45	31	47	41	95
**Veron L**	48	23	51	34	95

**Table 2:** Chemical composition, Hyp and estimated collagen content of raw materials used for lab-scale hydrolysis given (in g/100 g wet weight). Errors given in SD of duplicate measurements.

Element	CM (g/100 g)	TCT (g/100 g)	MDCR (g/100 g)
Protein	22 ± 0.2	31 ± 1	20 ± 1
Ash	1.2 ± 0	5.1 ± 0.8	1.8 ± 0.3
Fat	2.6 ± 0.07	4.6 ± 0.3	20 ± 0
Water	74 ± 0.2	59 ± 3	59 ± 0.4
Hyp	0.06 ± 0.01	3.05 ± 0.03	1.02 ± 0.01
Collagen *	0.44 ± 0.07	22.6 ± 0.2	7.6 ± 0.07

* Value calculated from assumption of 13.5% Hyp content of total amino acids in poultry collagen [25].

**Table 3:** The protein recovery from raw materials after 1 h of hydrolysis at the lab scale at 40 °C, at ambient pH, with the selected proteases on four different raw materials (errors given in SD of replicate hydrolysates).

	Protein Recovery (%)			
Protease	TCT	CM	TCT+CM	MDCR
Endocut-02	40.1 ± 4.2	36.7 ± 2.3	38.0 ± 2.1	52.6 ± 1.5
Bromelain	53.9 ± 4.7	48.4 ± 3.8	51.1 ± 3.0	55.1 ± 1.5

**Table 4:** The protein concentration (g/100 g) in dried hydrolysates resulting from Bromelain (B) and Endocut-02 (E) hydrolysis (Dumas nitrogen x 6.25) from samples taken at 40 and 50 min of hydrolysis, and from the final dried hydrolysate at 60 min (mean value of duplicate hydrolysate series). In the lower row, the difference in protein concentration between the 50 min sample and the 60 min hydrolysate.

	CM (g/100 g)		TCT (g/100 g)		TCM+CM (g/100 g)		MDCR (g/100 g)	
Time (min)	B	E	B	E	B	E	B	E
40	90	87	73	75	85	78	79	73
50	89	86	76	69	85	79	81	74
60	88	89	97	93	92	88	91	90
Δ (60–50)	−1	3	21	24	7	9	10	16

**Table 5:** The resulting protein yield (% of proteins liberated from start material), the protein concentration (g/100 g dry matter) in the 60 min sample taken during hydrolysis, and in the final product hydrolysate after MDCR hydrolysis (40 °C, 1 h). Hydrolysis was performed using either Endocut-02 or Bromelain, a combination of both for the full hour (BE), or adding either Bromelain or Endocut-02 30 min before adding the other protease (B+E or E+B) (errors given in SD of replicate hydrolysates). The Δ [protein] column shows the difference between the end product and 60 min sample protein concentrations.

	Protein Yield (%)	[Protein], 60 min Sample (g/100 g Dry Matter)	[Protein], End Product (g/100 g Dry Matter)	Δ [Protein] (g/100 g)
Endocut-02	54.5 ± 1.0	80.8 ± 3.2	97.7 ± 0.3	16.9
Bromelain	50.8 ± 3.6	75.1 ± 1.5	94.3 ± 1.8	19.2
BE	64.3 ± 4.3	85.0 ± 0.9	95.3 ± 0.2	10.3
B+E	69.3 ± 1.1	84.0 ± 0.8	95.5 ± 1.3	11.5
E+B	70.7 ± 5.3	85.1 ± 2.3	96.0 ± 0.3	10.9

**Table 6:** Protease information. The commercial name, vendor and properties of all proteases are included in the first azo-casein hydrolysis.

Protease	Vendor	Activity	Vendor Enzyme Description	pH	Temp
Alcalase	Novozymes	Endo/exo	Serine endopeptidase (mainly Subtilisin A)	7.0–10	30–70
Bromelain BR 1200	Bromelain Enzyme	Endo	Cysteine protease	4.0–9.0	40–65
Corolase 2TS	AB Enzymes	Endo	Thermolysin, extracellular neutral metalloprotease	6.0–9.0	up to 70
Corolase 7090	AB Enzymes	Endo	Bacillolysin metallo endopeptidase	6.5–7.5	45–70
Endocut-01	Tailorzyme	Endo	Neutral endo-protease	6.0–8.0	45–55
Endocut-02	Tailorzyme	Endo	Alkali endoprotease	(6)7.0–10	55–65
Endocut-03	Tailorzyme	Endo	Alkali endoprotease	7–10	55–70
Flavourzyme	Novozymes	Exo	Mix of different exopeptidases	5.0–7.0	35–65
FoodPro 30L	DuPont/Danisco	Endo	Alkaline serine endopeptidase	6.0–7.5	45–65
FoodPro 51 FP	DuPont/Danisco	Endo/exo	Mix of exo- and endopeptidases	8.0–10.0	45–60
FoodPro PNL	DuPont/Danisco	Endo	Neutral metallo endopeptidase	6.0–7.5	50–70
Protamex	Novozymes	Endo	Trypsin, bacillolysin, subtilisin	7.0–10	35–60
MaxiPro NPU	DSM	Endo	Neutral endo-protease	5.5–7.5	25–55
Neutrase	Novozymes	Endo	Neutral, zinc metallo endo-protease	5.5–7.5	45–55
PROMOD 144GL	Biocatalysts	Endo	Ultralow sulphite papain	5.0–7.5	50–70
PROMOD P950L	Biocatalysts	Endo	Microbial alternative to papain	5.0–7.0	50–60
TAIL-10	Tailorzyme	Endo	Alkaline serine endopeptidase, ficin, papain, pepsin	7.0–9.0	30–70
VERON L	AB Enzymes	Endo	Proteolytic enzyme preparation based on papain	5.0–7.5	50–70

## Data Availability

Data are available on request.

## Supplementary Materials

The following are available online. Figure S1: Weight-based yields, Figure S2: SEC chromatograms, Figure S3: SDS-PAGE results, Table S1: Results from curve-fitting azo-casein reactions, Table S2: SEC standards and calibration results.

## Sample Availability

Samples of the compounds are not available from the authors.
