# Supplementary material for: Exploring Effects of Protease Choice and Protease Combinations in Enzymatic Protein Hydrolysis of Poultry By-Products
Source: Molecules. 2021 Aug 31;26(17):5280. doi: 10.3390/molecules26175280 (PMC8434180; doi:10.3390/molecules26175280)
Supplement: Supplementary file 1 [file molecules-26-05280-s001.zip › molecules-1314144-supplementary.pdf]

Supplementary Information

Exploring Effects of Protease Choice and Protease Combinations in Enzymatic Protein Hydrolysis of Poultry by-Products

Diana Lindberg, Kenneth Aase Kristoffersen, Sileshi Gizachew Wubshet, Linn Maria Gundersen Hunnes,
Marte Dalsnes, Katinka Riiser Dankel, Vibeke Høst and Nils Kristian Afseth *

Nofima AS, Osloveien 1, 1433 Ås, Norway; Diana.Lindberg@Nofima.no (D.L.); Kenneth.Kristoffersen@Nofima.no (K.A.K.); Sileshi.Wubshet@Nofima.no (S.G.W.); linnmaria.g.hunnes@gmail.com (L.M.G.H.); m.dalsnes@gmail.com (M.D.); Katinka.Dankel@Nofima.no (K.R.D.); Vibeke.Host@Nofima.no (V.H.)
* Correspondence: Nils.Kristian.Afseth@Nofima.no; Tel.: +47-6497-0418

Table S1. Results from curve-fitting azo-casein reactions. The proteases included in proteolysis using the azo-casein assay (pH 7.0, 40 °C), the protease vendors, the resulting k, m and R^2 values from the straight-line curve fit, the value of x when y=1, and the corresponding dilution of the protease within the vial during proteolysis corresponding to that value.

**Protease**	**k**	**m**	**R^2**	**x when y = 1**	**Dilution (fold)**
Alcalase	4771	-0.1167	0.973	2.34E-04	4272
Bromelain BR 1200	9173	0.1005	0.994	9.81E-05	10198
Corolase 2TS	891	0.0040	0.994	1.12E-03	894
Corolase 7090	1515	-0.0214	0.998	6.74E-04	1483
Endocut-01	957	-0.0044	0.970	1.05E-03	953
Endocut-02	7998	0.2524	0.990	9.35E-05	10698
Endocut-03	6697	0.1040	0.995	1.34E-04	7474
Flavourzyme	1580	-0.0301	0.999	6.52E-04	1534
FoodPro 30L	6491	0.1039	0.989	1.38E-04	7244
FoodPro 51 FP	1196	0.0990	0.995	7.53E-04	1327
FoodPro PNL	1531	-0.0551	0.999	6.89E-04	1451
Protamex	1728	-0.0608	0.997	6.14E-04	1629
MaxiPro NPU	3838	-0.031	0.784	2.7 E-03	372
Neutrase	489.9	-0.037	0.989	2.1 E-03	472
Promod 144GL	1187	-0.0431	0.997	8.79E-04	1138
Promod P950L	4674	0.0569	0.986	2.02E-04	4956
Tail-10	9260	0.1241	0.997	9.46E-05	10572
Veron L	4050	-0.1343	0.934	2.80E-04	3570

Table S2. Retention times of calibration standards analyzed with a BioSep SEC s2000 column (Phenomenex) on an Agilent and a Thermo instrument. The compounds, their molecular weights (Mw), the logarithm of the molecular weights, the mean retention time (RT), and the standard deviation (SD) of the retention times from three replicate runs are presented. All standards were obtained from Sigma Aldrich (St. Louis, MO).

Calibration standard	Mw (g/mol)	Log10(Mw)	Mean RT, Agilent	SD, Agilent	Mean RT, Thermo	SD, Thermo
Bovine Serum Albumin	66000	4,820	5,954	0,005	5,954	0,005
Albumin from chicken egg white	44287	4,646	6,004	0,005	6,004	0,005
Carbonic anhydrase	29000	4,462	6,024	0,000	6,024	0,000
Lysozyme	14300	4,155	6,490	0,008	6,490	0,008
Cytochrome c from bovine heart	12327	4,091	6,229	0,005	6,229	0,005
Aprotinin from bovine lung	6511	3,814	6,993	0,005	6,993	0,005
Insulin Chain B Oxidized from bovine pancreas	3496	3,544	8,557	0,000	8,557	0,000
Renin Substrate Tetradecapeptide porcine	1759	3,245	8,243	0,005	8,243	0,005
Angiotensin II human	1046	3,020	8,821	0,005	8,821	0,005
Bradykinin Fragment 1-7	757	2,879	9,335	0,005	9,335	0,005
[D-Ala2]-Leucine enkephalin	570	2,756	11,379	0,005	11,379	0,005
Val-Tyr-Val	379	2,579	10,985	0,005	10,985	0,005
L-Tryptophan	204	2,310	11,837	0,005	11,837	0,005

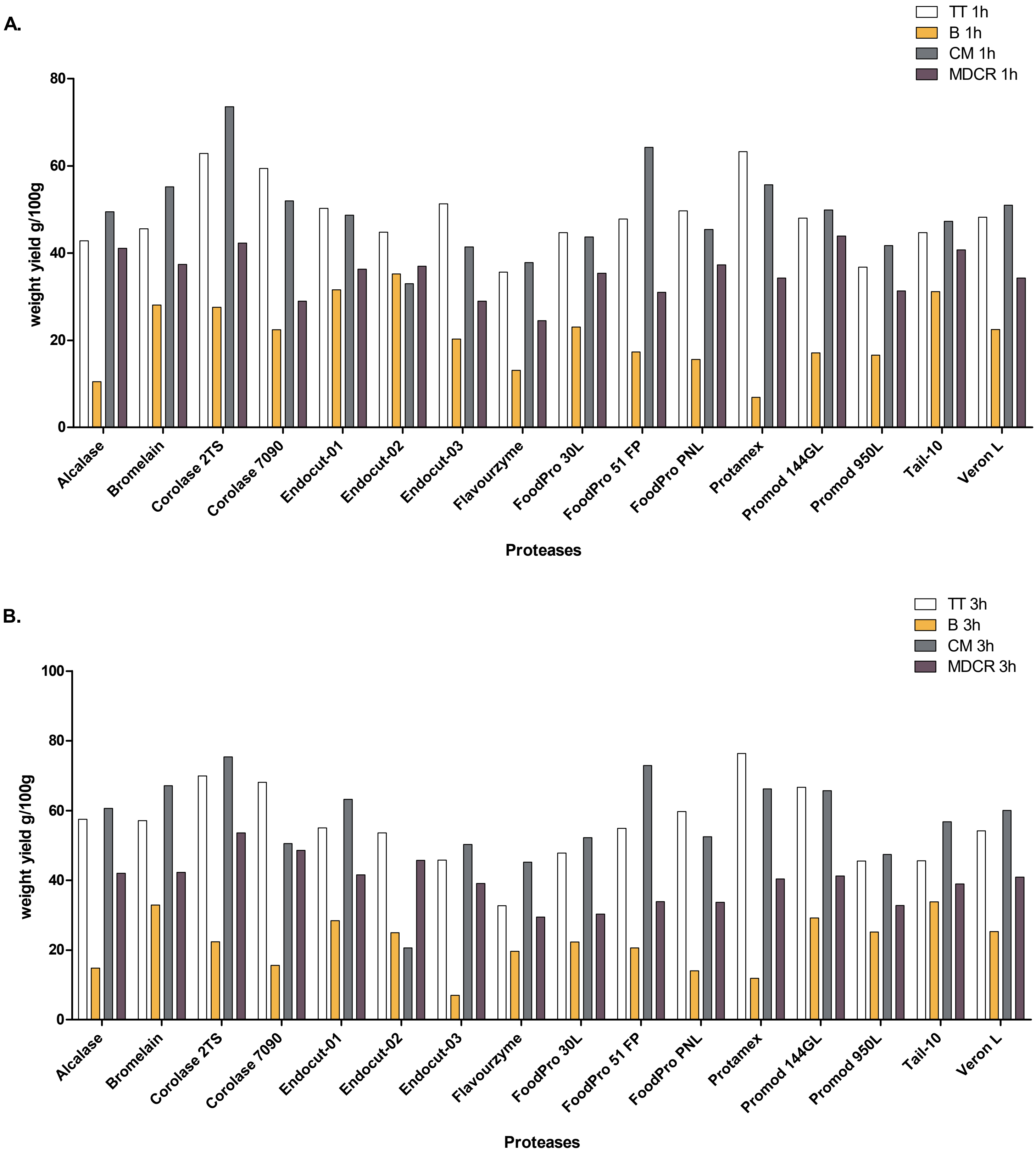

Figure S1. Weight-based yields. The resulting weight-based yield after hydrolysis of 2 g of four different materials, TT (white), B (yellow), CM (grey), and MDCR (dark purple) after A) 1 h, or B) 3 h at 40 °C in 0.01 M sodium phosphate buffer in an end-over-end apparatus.

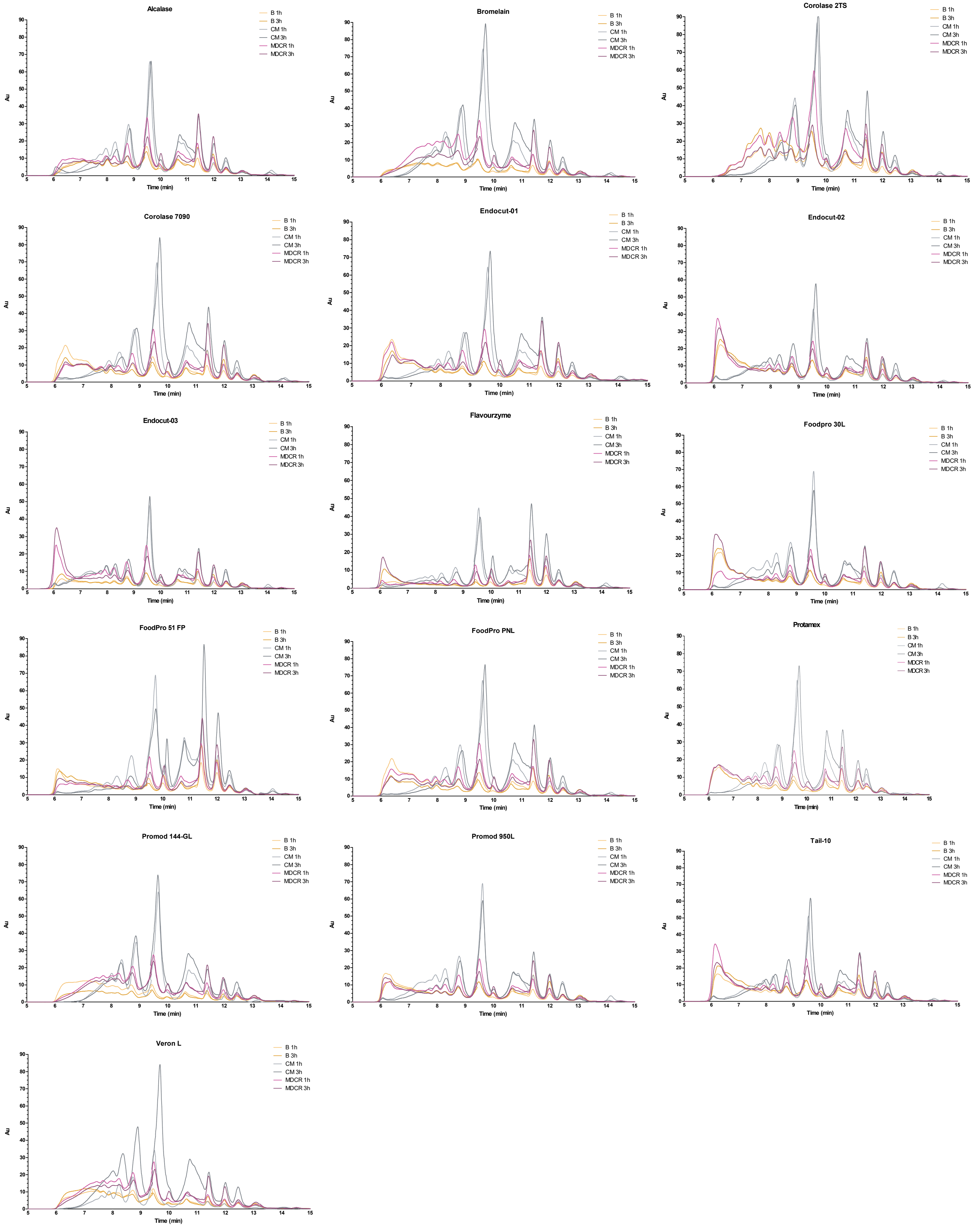

Figure S2. The SECchromatograms resulting from hydrolysis of 16 proteases on bones (B, in yellow), chicken meat (CM, in grey) and MDCM (in purple) after 1 and 3 hours, with 1h. chromaograms in a lighter shade of each color.

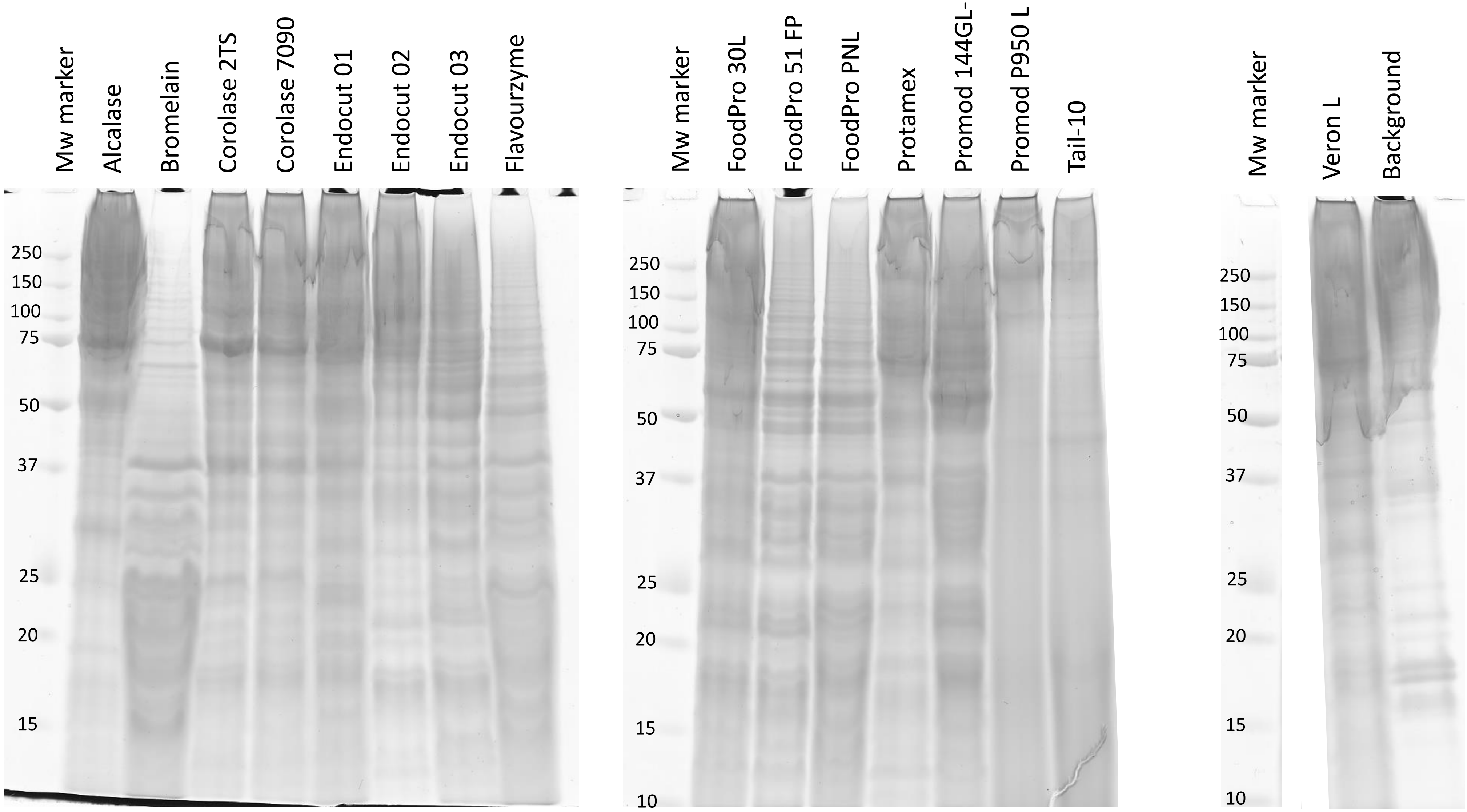

Figure S3. The resulting three SDS PAGE gels after running turkey tendon hydrolysates from the 16 proteases of the small-scale raw material screening as well as a background sample, without added protease. The first lane in each gel, "Mw marker", is containing the molecular standard sample with molecular weights as defined in the figure. The last lane has been cropped from the gel in the middle, as well as lanes 2-7 from the gel to the right (Mw marker in lane 1 and Veron L and Background in lanes 8-9).
